# A Narrative Review on Biologic Joint Therapy in Chronic Bilateral Mandibular Luxation

**DOI:** 10.31661/gmj.v13iSP1.3645

**Published:** 2024-12-25

**Authors:** Seyed Hossein Shahidi, Sadaf Ghasemzadeh, Sayeh Zarehgar, Nader Ghotbi, Fatemeh Bagherian, Reza Mahmoudi Anzabi, Meysam Mohammadikhah, Nima Ahangar Atashi

**Affiliations:** ^1^ Department of Oral and Maxillo facial surgery, School of Dentistry, Islamic Azad University of Shiraz, Shiraz, Iran; ^2^ Department of Oral Maxillofacial Surgery, Faculty of Dentistry, Tabriz University of Medical Science, Tabriz, Iran; ^3^ Department of Prosthodontics, School of Dentistry, Unciano colleges and general hospital, Manila, Philipines; ^4^ Isfahan Azad university, School of Dentistry, Isfahan, Iran; ^5^ Moscow State University of Medicine and Dentistry, Moscow, Russia; ^6^ Department of Orthodontics, Faculty of Dentistry, Tabriz University of Medical Sciences, Tabriz, Iran; ^7^ Department of Oral and Maxillofacial Surgery, School of Dentistry, Alborz University of Medical Sciences, Karaj, Alborz, Iran; ^8^ Department of Oral and Maxillofacial Surgery, Faculty of Dentistry, Tabriz University of Medical Science, Tabriz, Iran GMJ.2024;13:e3645 www.salviapub.com Correspondence

**Keywords:** Temporomandibular Joint, Luxation, Dislocation, Platelet-rich Plasma, Hyaluronic Acid, Autologous Blood Injection

## Abstract

**Background:**

Temporomandibular joint (TMJ) luxation, or dislocation, is a relatively infrequent but highly consequential condition that requires prompt medical attention. This narrative review strives to offer a thorough summary of TMJ luxation treatment modalities, with a focus on recent advancements in biological treatments.

**Materials and Methods:**

A narrative review was conducted by synthesizing data from various sources, including systematic reviews, meta-analyses, and clinical trials. The review covers studies published up to 2024.

**Results:**

Various surgical techniques, such as eminectomy, midline mandibulotomy, and extended prosthetic total TMJ reconstruction (eTMJR), have been used to treat chronic TMJ dislocation. Platelet-rich plasma (PRP) and hyaluronic acid (HA) have shown promise in treating TMJ disorders. PRP can promote healing and reduce inflammation, while HA improves mandibular mobility and reduces pain. A combination of HA and PRP has been found to be particularly effective in treating TMJ disorders. Autologous blood injection (ABI) has been shown to be effective in reducing the recurrence of dislocation and improving patient outcomes, with fewer side effects compared to other treatments.

**Conclusion:**

TMJ luxation is a multifactorial condition that requires prompt and appropriate management to prevent long-term complications. While various surgical techniques are available, biological treatments such as PRP, HA, and ABI have shown promising results, particularly in reducing pain and improving joint function. Further research, including randomized controlled trials, is required to ascertain the most efficacious therapy modalities and to refine clinical guidelines for the management of TMJ luxation.

## Introduction

Temporomandibular joint (TMJ) luxation, or dislocation, is an infrequent incident that substantially impacts the affected individual and generally requires prompt medical attention [1]. Additionally, the field of dentistry regards TMJ luxation as a highly consequential condition due to its substantial psychological and social ramifications [2]. By classification, TMJ hypermobility may manifest as either a luxation or a subluxation. A TMJ subluxation occurs when the condyle moves past the articular eminence. At the same time, the jaw is open. It stays still for a short time before returning to its original position in the fossa, which can occur spontaneously or require the patient to manipulate the joint manually [1][2]. In 2014, Research Diagnostic Criteria (RDC/TMD) proposed a classification system for Temporomandibular Disorders (TMD) to include less common but significant clinical disorders. TMJ dislocation refers to the misalignment of the joint where the mandibular condyle no longer fits properly within the temporal bone’s glenoid fossa, or the condyle’s head moves out of its usual position in the fossa [3][4][5].

Peak incidence of TMJ dislocation occurs in individuals aged in early adulthood, with a mean duration of 7.9 weeks [6]. A systematic quantitative synthesis of evidence study assessed the frequency of TMJ across different age groups, finding an overall prevalence of about 31% in adults and elderly individuals and 11% in children and adolescents [7]. incidence of TMJ dislocation in Germany is estimated to be at least 25 per 100,000 per year [8]. A retrospective study of 17 cases of TMJ dislocation treated surgically found that 9 cases were recurrent dislocations [9]. Several risk factors have been identified, including hypermobility, eminectomy, and chronic protracted mandibular dislocation [10]. The aetiology of TMJ dislocation is multifactorial, with excessive mouth opening while yawning being a common cause [11]. Medical intervention for persistent TMJ dislocation is frequently essential to reestablish oral functionality and enhance the standard of living. Research on the management of chronic recurring dislocations using an adjusted eminoplasty method documented favorable outcomes in boosting patient well-being [12]. Diverse surgical methods have been employed to address individuals with chronic TMJ dislocation, such as eminectomy, which has been utilized with commendable results [13][14]. The LeClerc technique, enhanced by Gosserez & Dautrey, has also been applied to manage chronic recurrent anterior dislocation of the TMJ [15]. A subsequent evaluation of 12 patients treated with this technique revealed no reappearance of dislocation over a monitoring duration of up to 5 years and 3 months [15]. The Dautrey technique has also been utilized to treat 12 patients with chronic recurrent dislocation of the TMJ, with no recurrence in 11 patients over a monitoring duration of up to 5 years and 2 months [16].

Additional studies have detailed the application of various surgical approaches, including midline mandibulotomy [17] and extended artificial complete TMJ reconstruction [18]. An organized review of the sources on surgical treatment of repetitive TMJ dislocation discovered that various surgical techniques have been used, but there is a need for further research to determine the most effective treatment [19]. There are also other techniques like dextrose prolotherapy that clinical outcomes show limited but positive evidence, with improvements in joint pain, maximum mouth opening, and patient satisfaction, based on a 2023 review [20]. but yet evidence is scare in form of high-quality studies like randomized trials. The realm of biologic treatments for mandibular luxation has garnered significant attention in recent years, with a plethora of studies delving into the intricacies of this complex condition [21].

The study of bone and immune system interactions in mouth and jaw conditions has become a central area of investigation, with practical applications derived from biological processes being examined [21]. Additionally, tissue reconstruction has arisen as a hopeful approach for managing temporomandibular joint issues, with studies investigating the use of biomaterials and stem cells to repair damaged tissues [22]. As the current literature on TMJ luxation is plagued by a lack of comprehensive and standardized treatment protocols, and existing surgical techniques often yield inconsistent results and may be associated with significant morbidity, we aimed at providing a comprehensive overview of the recent advancements in biological treatments for this condition. Furthermore, the scarcity of high-quality studies, such as randomized controlled trials, and the limited evidence on the efficacy of biologic joint therapy in chronic bilateral mandibular luxation, underscores the need for further research in this area. This study is novel in that it focuses on the emerging field of biologic treatments, including PRP, HA, ABI, and Botox, which have shown promising results in reducing pain and improving joint function, and strives to add to the advancement of more efficient and minimally invasive treatment modalities for TMJ luxation.

## Concentrated Platelet Therapy and Hyaluronic Gel

Studies have demonstrated that PRP can be an effective adjunctive treatment for TMJ disorders, including luxation [23][24]. The injection of PRP into the TMJ has been shown to promote healing and reduce inflammation, thereby alleviating symptoms associated with luxation [23][24]. Furthermore, the use of PRP in combination with other treatment modalities, such as intermaxillary fixation, has been found to be effective in managing recurrent TMJ luxation [23][24]. However, Additional investigation is required to thoroughly clarify the effectiveness of PRP in the management of mandibular dislocation [24]. Chęciński et al. (2024) conducted a controlled clinical trial comparing HA and PRP in 78 patients with temporomandibular joint disorders. The study found that HA significantly improved mandibular mobility, while PRP did not show statistically significant changes. Notably, HA outperformed PRP in improving abduction and protrusion, but not in lateral movements [25].

In a systematic review by Chęciński et al. (2024), the combination of HA and PRP was evaluated for its efficacy in treating TMJ disorders. The review included six studies and found that the HA/PRP mixture significantly improved mandibular abduction by 2.52 mm and reduced pain by −1.33 at the 6-month follow-up. This suggests that the HA/PRP combination is particularly effective in cases where HA or PRP alone may not be sufficient [26].

Sane et al. (2024) compared the efficacy of in bone restoration following the operative extraction of embedded lower jaw third molars. The investigation discovered no notable variations in bone density or quantity between PRP and PRF at 1- and 4-months post-surgery. This indicates that both PRP and PRF are effective in bone regeneration, with PRF being a potentially preferable option due to its ease of preparation [27].

Aishwarya and Rai (2024) investigated the utilization of injectable platelet-rich fibrin (I-PRF) in treating TMJ hypermobility. The study involved 26 patients who received a single I-PRF injection. Results showed significant reductions in maximal interincisal mouth opening, locking episodes, pain scores, and joint sounds during a 90-day monitoring timeframe. This suggests that I-PRF is an uncomplicated, slightly intrusive, and budget-friendly treatment for TMJ hypermobility [28].

Alsaadi et al. (2024) evaluated the effects of sodium hyaluronic acid (HA) and a chitosan-hyaluronate hybrid gel (CH) in managing frontal disc displacement not using reduction. The study found that both treatments significantly reduced pain and improved mandibular mobility, but the CH group showed better outcomes regarding greatest oral aperture and pain reduction at 1 and 3 months. This indicates that the CH gel is more potent than HA solely in managing anterior disc displacement without restoration [29].

Some studies are suggesting that HA may have a beneficial effect on joint mobility and pain reduction [30][31]. Nevertheless, a comparative analysis of HA and PRP injections revealed that PRP may be more efficient in alleviating discomfort in individuals with TMJ osteoarthritis, particularly at 12 months post-injection [31].

The use of HA in treating TMJ disorders has been explored in several studies, with some researchers suggesting that it may improve joint biomechanics and reduce pain [30][32]. However, the exact mechanisms by which HA exerts its effects on the TMJ are not fully understood and require further investigation [33]. Additionally, the comparison between HA and PRP injections has yielded mixed results, with some studies suggesting that PRP might be more efficient in diminishing discomfort and enhancing joint mobility [31]. Additional investigation is required to completely clarify the therapeutic potential of HA and PRP in managing TMJ disorders and to determine the optimal treatment approach for affected individuals [30][31][32][33].

## Autologous Blood Injection

In a comprehensive evaluation of various treatments for chronic recurrent TMJ dislocation, several studies have compared different techniques, including autologous blood injection (ABI), eminectomy, and botulinum toxin type A (BTX-A) injections. One study randomly divided 20 patients into two groups, with one group receiving ABI and the other undergoing eminectomy. Both groups showed significant initial improvements in parameters such as episodes of subluxation per week, joint sounds, pain, and maximal incisal opening. However, long-term follow-up revealed the onset of osteoarthritic changes in the eminectomy group, as detected by digital radiographic imaging [34].

Another study, published in the Journal of Maxillofacial and Oral Surgery (2024), compared ABI with dextrose prolotherapy in 32 patients. Both groups showed comparable reductions in pain levels and joint hypermobility within a week. However, at half-yearly and yearly check-ins, the ABI group demonstrated significantly lower mouth opening and fewer dislocations [35].

A third study, conducted by Noha and Wessam (2024), compared ABI with BTX-A injections in 20 patients with chronic TMJ dislocation. Both groups showed no dislocations on clinical examination post-injection, and MRI scans confirmed the absence of dislocations in all patients. One-month post-injection, the ABI group demonstrated a notable decrease in TMJ dislocation and pain associated with the movement of the lateral pterygoid muscle (LPM) [36].

This technique involves the injection of the patient’s own blood into the affected joint, with the aim of promoting healing and reducing the recurrence of dislocation. Studies have shown that autologous blood injection can be was a secure and efficient therapeutic approach for chronic presistant temporomandibular joint dislocation, with some research indicating that it can reduce the frequency of dislocation and enhance patient results [37][38][39][40]. The histopathologic effects of autologous blood injection on the temporomandibular joint have also been investigated, with one study finding that it does not modify the joint structure in animal model of rabbits [40]. Additionally, comparisons have been made between autologous blood injection and other treatment modalities, such as dextrose prolotherapy, with some research suggesting that autologous blood injection may be a more effective treatment option [41].

The use of ABI for the management of mandibular luxation has been supported by several studies, which have demonstrated its efficacy in reducing the recurrence of dislocation and improving patient outcomes [37][39]. For example, a prospective, randomized, controlled clinical trial discovered that ABI, either alone or in combination with intermaxillary fixation, was effective in treating persistent repetitive misalignment of the jaw joint [39]. Additional research discovered that autologous blood injection was linked to a notable decrease in the occurrence of dislocation and enhancement in patient symptoms [38]. Furthermore, review research on self-derived blood injection for the management of jaw joint dislocation discovered that it was a secure and efficient therapeutic approach [40]. Overall, these studies indicate that while all treatments provide initial clinical benefits, ABI emerges as a superior and more conservative approach with fewer side effects and better long-term outcomes in managing chronic recurrent TMJ dislocation.

## Botulinum Toxin

According to new research, most researchers agree that using botulinum toxin in the LP muscle has shown promising results in easing the symptoms of TMD and muscle dystonia [41][42][43][44][45][46][47]. Relaxation and maintaining a consistent muscle tone could alleviate the pain associated with TMJ clicks, which may stem from an overly tight lateral pterygoid muscle relative to the articular disc [48][49]. Using BTX-A to treat LP muscle hyperactivity has been a longstanding practice. The therapeutic uses of botulinum toxin encompass a range of procedures, including dental implant surgeries, correction of gummy smiles, management of headaches (including migraines and trigeminal neuralgia), and injection into the masseter muscle to treat temporomandibular disorders. Research has shown that botulinum toxin injections into the lateral pterygoid muscles can provide a predictable and prolonged period without renewed dislocation [50]. A study conducted on 21 patients with recurrent temporomandibular joint dislocation found that only two patients suffered further dislocation within the study period of 6 months to 3 years [50].

Another study reported the successful treatment of five cases of dislocation in elderly patients with neurological or other severe systemic disease using botulinum toxin type A injections into the lateral pterygoid muscles [51]. The use of incobotulinumtoxinA in refractory temporomandibular disorder due to disk dislocation has also been evaluated, with results indicating that botulinum toxin injection of the masticatory muscles is safe and effective in reducing pain [52]. Furthermore, botulinum toxin treatment has been shown to be effective in treating neurogenic dislocation of the temporomandibular joint, with few side effects [53]. However, there is a need for randomized controlled trials to further investigate the efficacy of Botox in treating mandibular dislocation.

## Conclusion

The review places a significant emphasis on organic therapies such as Hyaluronic Acid (HA), Autologous Blood Injection (ABI), and Platelet-Rich Plasma (PRP) which have emerged as promising alternatives to traditional surgical interventions. These treatments leverage the body’s natural healing mechanisms to promote joint repair and reduce inflammation. The review includes a comparative analysis of biologic treatments, highlighting the relative strengths and limitations of each. For instance, while HA has shown significant benefits in improving mandibular mobility and reducing pain, PRP may be more efficient in diminishing discomfort in individuals with TMJ osteoarthritis. ABI, on the other hand, has emerged as a superior and more conservative approach with fewer side effects and better long-term outcomes. The scarcity of high-quality studies, such as randomized controlled trials, underscores the need for further research to establish the most effective treatment modalities and to refine clinical guidelines. The review demands for a uniform method to the handling of TMJ dislocation, highlighting the significance of personalized care strategies that take into account the particular requirements and circumstances of every patient.

## Conflict of Interest

None declared.
